# Valsartan vs. other angiotensin II receptor blockers in the treatment of hypertension: a meta-analytical approach

**DOI:** 10.1111/j.1742-1241.2009.02028.x

**Published:** 2009-05

**Authors:** R M Nixon, E Müller, A Lowy, H Falvey

**Affiliations:** 1 Novartis Pharma AGBasel, Switzerland; 2 Analytica InternationalLoerrach, Germany

## Abstract

**Objective:**

To compare the efficacy of valsartan in systolic (SBP) and diastolic blood pressure (DBP) reduction with other angiotensin II receptor blockers (ARBs) in essential hypertension.

**Methods:**

Systematic literature search of databases between October 1997 and May 2008. Meta-analysis of short-term, double-blind, parallel group, randomised controlled trials (RCTs) for treatment of adult hypertension (DBP: 90–115 mmHg). Random-effects meta-regression adjusting for baseline blood pressure (BP) was used to analyse the data. Mean change in SBP and DBP was estimated for each individual drug and dose combination.

**Results:**

In all, 31 RCTs (*n* = 13,110 patients) were included in the analysis. Six studies include trial arms with candesartan, six irbesartan, 13 losartan, two olmesartan, five telmisartan and 12 valsartan. The weighted average reduction in mean SBP and DBP for valsartan 160 mg was −15.32 mmHg (95% CI: −17.09, −13.63) and −11.3 mmHg (95% CI: −12.15, −10.52) and for 320 mg was −15.85 mmHg (95% CI: −17.60, −14.12) and −11.97 mmHg (95% CI: −12.81, −11.16); these are statistically significantly greater reductions compared with losartan 100 mg, which was −12.01 mmHg (95% CI: −13.78, −10.25) and −9.37 mmHg (95% CI: −10.18, −8.54) for SBP and DBP respectively. There is evidence that valsartan 160 mg reduces SBP and DBP more than irbesartan 150 mg and reduced DBP more than candesartan 16 mg. No other statistically significant difference in efficacy is demonstrated.

**Conclusion:**

Valsartan administered at 160 or 320 mg is more effective at lowering BP than losartan 100 mg and shows comparable efficacy to other ARBs in patients with essential hypertension.

**Review Criteria**

Data was gathered from prospective double-blind randomised controlled trials, with at least one ARBs monotherapy arm with no or forced titration. Studies had to report change in office systolic or diastolic blood pressure from baseline to follow-up six to 12 weeks later. A random-effect meta-regression model was used to estimate the overall mean change in blood pressure from baseline to follow-up.

**Message for the Clinic**

Previous meta-analyses have demonstrated that ARBs have comparable efficacy. However, none have included valsartan at 160 and 320 mg. This paper shows that valsartan at doses of 160 mg or 320 mg is more effective at lowering blood pressure than losartan 100 mg. For other ARBs at comparable doses, valsartan achieves comparable antihypertensive efficacy. Valsartan has a strong dose–response relationship when increasing from 80 mg to 160 mg or 320 mg.

## Introduction

Hypertension currently affects approximately one billion adults globally. It is a major risk factor for cardiovascular diseases (CV) and stroke and is associated with metabolic syndromes including insulin resistance and lipid abnormalities. The high prevalence of hypertension has contributed to the present pandemic of CV disease, which now accounts for 30% of all deaths worldwide (1). As the population ages and the prevalence of contributing factors such as obesity, sedentary lifestyle and smoking rise, this figure is projected to increase by 60% to 1.56 billion by the year 2025 (1,2). The risk of hypertension increases with age and is associated with gender and ethnicity. The morbidity and mortality associated with uncontrolled hypertension result in a substantial economic burden as a result of drug costs, hospitalisations, surgery and other healthcare resources. This cost is compounded by the humanistic burden and effect on quality of life associated with lifestyle modifying adverse events. Despite global awareness of hypertension, its consequences and the availability of effective therapeutics, an estimated 32% of hypertensive patients remain untreated (3). The global proliferation of cost effective, tolerable long-term therapy is paramount for reducing this growing catastrophe.

### Renin-angiotensin-aldosterone-system and the role of ARBs

The renin-angiotensin-aldosterone-system (RAAS) plays an integral role in the pathophysiology of hypertension, functioning as a primary regulator in the control of fluid volume, electrolyte balance and blood volume. In conjunction, angiotensin II causes potent vasoconstriction, aldosterone secretion and sympathetic activation, all of which contribute to the development of hypertension. Angiotensin II receptor blockers (ARBs) modulate the RAAS system by blocking the activation of angiotensin II AT_1_ receptors resulting in, among other effects, vasodilatation, reduced secretion of vasopressin and reduced production and secretion of aldosterone.

There are currently six ARBs used as first line treatment in hypertension: valsartan, candesartan, irbesartan, losartan, olmesartan and telmisartan. As the first ARBs were introduced in the mid-1990s, numerous clinical trials have been conducted to evaluate their efficacy and tolerability. Concerning valsartan, more than 34,000 patients with hypertension and its complications have been included in extensive clinical trials such as the Val-HeFT (4), VALIANT (5) and VALUE (6) trials.

Valsartan is a non-peptide, orally active and specific angiotensin II antagonist, which demonstrates high affinity to the AT1 receptor subtype. Although widely used in the control of hypertension, its use at higher dose is less widespread. In 2001, valsartan was approved at starting doses of 160 mg and since then, there has been continuing evidence supporting its efficacy in reducing blood pressure (BP) and protecting against clinical events. Studies demonstrate that the placebo-like tolerability and once daily dosing schedule of valsartan result in improved patient compliance and treatment persistence, resulting in increased drug efficacy (7,8). Furthermore, this tolerability has been found to be stable over a wide dosing range (9). These advantages, in addition to the comparative cost-effectiveness of valsartan, mean that it remains a favourable option for long-term control of adult hypertension (10).

### Dose–response effect: the need for further analysis

Integrated analysis of valsartan has demonstrated clear dose-dependent efficacy and ability to achieve BP goals at doses of 160–320 mg (11); however, there is a notable absence of head-to-head trials comparing valsartan dose 320 mg with other ARBs. The only study to date is a recent publication by Giles et al. (12) comparing the efficacy of valsartan, olmesartan medoxomil and losartan potassium in a 12-week, forced titration randomised control trial. Results of this study demonstrate a dose effect throughout: At treatment week 4, reduction in seated DBP (SeDBP) is −9.2 mmHg for valsartan 80 mg. At week 8, this reduction in SeDBP increases to −11.6 mmHg for valsartan 160 mg. At week 12, there is a further increase to −12.4 mmHg for valsartan 320 mg (p < 0.05 vs. placebo). These results confirm that use of valsartan at 160 and 320 mg improve BP control.

Results encourage further comparisons at doses of 320 mg. Lack of other head-to-head trials motivates the need for indirect comparison. In the absence of said trials, meta-analysis is useful for comparing ARBs at a range of dosing options. No meta-analysis to date has compared high-dose (320 mg) valsartan with other ARBs. Hence, the purpose of this meta-analysis is to compare high-dose valsartan with other ARBs in short-term, monotherapy trials with none or forced titration.

## Methods

### Literature search

A computerised systematic literature search was conducted using the following databases: MEDLINE, EMBASE, EMBASE Alert, *Cochrane Database of Systematic Reviews*, Cochrane Central Register of Controlled Trials and Science Citation Index (SciSearch). Both English and German randomised control trials were searched for, which were published between October 1997 and May 2008.

### Study selection

The following inclusion criteria were applied: prospective double-blind randomised controlled trials (RCTs); with at least one ARBs monotherapy arm with no or forced titration; studies recruiting patients representative of the general hypertension population (i.e. adults over 18 years, diagnosed with mild/moderate essential hypertension DBP: 90–115 mmHg). Office BP measured by automatic or cuff mercury sphygmomanometer, with measurements of (i) baseline and follow-up diastolic BP (DBP)/systolic BP (SBP) or (ii) baseline and change in baseline DBP/SBP. The following exclusion criteria were applied: patients with secondary hypertension, or CV (except diabetes, left ventricular hypertrophy and cardiomegaly); studies not reporting withdrawals; open-label; cross-over; titration to effect and ambulatory BP monitoring measurement trials. Studies with unacceptable methods of randomised allocation, double blinding and reporting of withdrawals were excluded. Table 1 shows drugs and dosages considered, and which doses are considered comparable between drugs.

**Table 1:** Doses per day of ARB therapy included in the meta-analysis

Treatment (mg/day)	Low dose	Medium dose	High dose
Candesartan	8	16	32
Irbesartan	–	150	300
Losartan	50	100	–
Olmesartan	10	20	40
Telmisartan	–	40	80
Valsartan	80	160	320

Doses are categorised as low, medium or high and are compared with one another within doses. In addition, valsartan 80 mg/day vs. irbesartan 150 mg/day and valsartan 320 mg/day vs. losartan 100 mg/day have been compared. ARB, angiotensin II receptor blocker.

### Validity assessment and data abstraction

Two independent reviewers completed all phases of literature selection, review and data abstraction. Discrepancies were resolved by third party consensus. Selected studies were quality assessed using a quality assessment tool in accordance with Cochrane Specifications (13) (see Appendix S1). Data were abstracted to a customised data extraction sheet which was performed by two reviewers and cross-checked for consistency.

For the meta-analytical models, four parameters were required for each treatment arm and each of SBP and DBP: the estimate of the mean change in BP from baseline to follow-up; the SD of this change; the estimate of the mean BP at baseline and the number of patients randomised. For inclusion in the meta-analysis, follow-up must be between 6 and 12 weeks. Where more than one result is available in this period, from interim analysis, the latest result has been used. The analysis is performed by dose. In the case of forced titration studies, the dose is taken as the maximum dose the patient was titrated to, rather than the starting dose.

### Quantitative data synthesis

**Figure 1:**
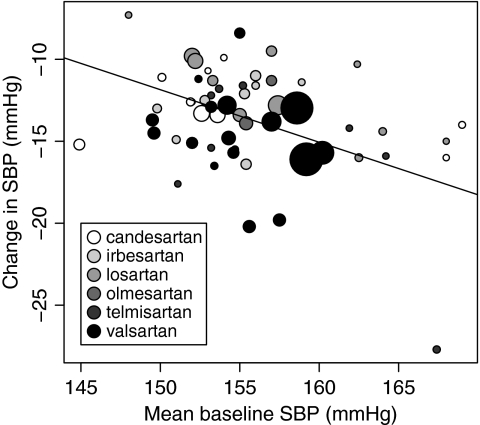
Observed mean baseline SBP all treatment arms plotted against the change in SBP. The linear regression weighted by the inverse of the variance of the change in SBP is also shown. The area of each circle is inversely proportional to this variance

A random-effect meta-regression model was used to estimate the overall mean change in SBP and DBP from baseline to follow-up. This model adjusts the estimate of the overall mean change in BP for the baseline BP. Figure 1 illustrates the relationship between baseline BP and change in BP, clearly showing that the reduction in BP is higher in trials with patients with higher BP at baseline. The model estimates the treatment effect by drug and dose. Full details of these models are given in the Appendix.

### Missing data imputation

Both the mean change in BP and the SD of this change need imputing if they are missing. Baseline BP data are complete as this is a requirement for study selection. Missing mean change in BP was imputed as the difference between mean follow-up and mean baseline BP. When reported, the change from baseline BP is commonly the complete case outcome. When missing, outcomes on all randomised patients were reported at baseline and the complete cases reported at follow-up, so imputing missing values in this way is assuming non-informative drop out. Missing SD of the change from baseline outcome was imputed from the reported SEM or the confidence intervals. If these were not given, then it was imputed using an imputation model, which uses the mathematical relationship between the SD at baseline, the SD at follow-up and the SD of the change in BP. Data from all the trial arms were used for imputing the missing SD, even if they contained treatments other than ARBs, doses not included in the analysis or elective titration arms. Details of this imputation are given in the Appendix.

## Results

### Trial flow

**Figure 2:**
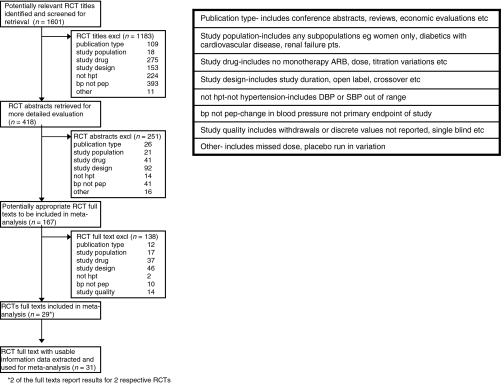
Trial flow diagram of the literature search resulting in 31 RCTs data extracted

Figure 2 shows the trial flow of selection stages for studies considered for inclusion in the meta-analysis. From a total of 1601 RCT titles for the publication period, 418 abstracts were reviewed, 251 of which were excluded. The most commonly excluded studies failed to meet the patient population inclusion criteria and hypertension thresholds. From full-text appraisal, further 138 studies were excluded predominantly for study type (open-label, cross-over), study duration (> 12 weeks), subpopulations (e.g. diabetic, renal disease and hyperlipidaemia) and measurement method (ambulatory BP measurement only). Of the resultant 29 full-text reports, two contained the results of two respective RCTs, resulting in 31 data extracted RCTs (*n*= 13,110), which were included for meta-analysis.

### Study characteristics

Summary information from the treatment arms of the RCTs abstracted is given in Table 2.

**Table 2:** Study characteristics and summary data extracted from the studies included in the analysis

						SBP			DBP			
Drug	Final dose	Titration type	*n*	Mean age (years)	Prop. male	Baseline	Change	SD of change	Baseline	Change	SD of change	References
Candesartan	2	None	59	54	0.49	152	−8.9	14.7	99	−7.1	8.82	Reif et al. (18)*
	4	None	63	55	0.7	152	−10.5	13.97	100	−8.4	8.3	Reif et al. (18)*
	8	None	82	60	0.57	169	−14	NA	102	−9	NA	Andersson and Neldam (19)
			60	55	0.57	154	−9.9	14.03	101	−8.7	8.5	Reif et al. (18)
	16	None	84	59	0.67	168	−16	NA	103	−10	NA	Andersson and Neldam (19)
			60	55	0.63	153	−10.7	14.82	100	−7.8	8.89	Reif et al. (18)
		Forced	91	54	0.62	150.1	−11.1	NA	100.2	−9.4	NA	Zuschke et al. (20)
			94	53	0.61	151.9	−12.6	NA	99.8	−10.3	NA	Zuschke et al. (20)
	32	None	59	55	0.7	152	−12.6	14.5	100	−10.2	8.43	Reif et al. (18)
		Forced	332	54.2	0.578	152.6	−13.3	NA	100.1	−10.9	NA	Bakris et al. (21)
			123	53	0.53	144.9	−15.2	11.88	94.2	−10.2	7.64	Kloner et al. (22)
			307	55.5	0.583	153.6	−13.4	NA	100.4	−10.5	NA	Vidt et al. (23)
Irbesartan	37.5	None	40	55	0.65	151	−7.5	10.5	100	−7.1	6.7	Kochar et al. (24)*
	75	None	55	56.7	0.67	157	−6.6	11.79	101.4	−6.1	7.56	Fogari et al. (25)*
	100	None	36	55	0.65	151	−11.1	12.5	100	−9.1	8.9	Kochar et al. (24)*
			79	52.8	0.69	149.8	−10.5	11.73	100.7	−9.7	7.55	Pool et al. study 2 (11)*
	150	None	53	54.6	0.6	158.9	−11.4	12.38	101	−8.3	7.86	Fogari et al. (25)
			57	54.1	0.63	156	−11.6	11.63	101.3	−9.7	7.4	Fogari et al. (25)
			134	56.1	0.493	152.8	−12.5	14.01	99.4	−8.88	8.57	Gradman et al. (26)
			142	53.1	0.54	155.3	−12.1	13.7	101.1	−9.7	7.75	Kassler-Taub et al. (27)
			145	51.9	0.586	156	−11	NA	104	−9.9	NA	Oparil et al. (28)
	200	None	75	52.8	0.69	149.8	−10.1	11.52	100.7	−9.8	7.36	Pool et al. study 2 (11)*
	300	None	140	55.6	0.57	155.4	−16.4	13.37	100.4	−11.7	7.57	Kassler-Taub et al. (27)
			43	55	0.65	151	−14.9	9.5	100	−10.2	5.8	Kochar et al. (24)
			78	52.8	0.69	149.8	−13	11.75	100.7	−11.6	7.51	Pool et al. study 2 (11)
Losartan	50	None	83	51.8	0.56	164	−14.4	NA	113.6	−10.6	NA	Ali et al. (29)
			83	59	0.57	168	−15	NA	104	−9	NA	Andersson and Neldam (19)
			127	53.2	0.65	NA	NA	NA	102.2	−7.9	9	Ikeda et al. (30)
			57	56	0.58	162.4	−10.3	13.59	100.7	−6	7.55	Mallion et al. (31)
			93	54.6	0.42	NA	NA	NA	100.8	−9.1	7.6	Monterroso et al. (32)
			146	51.6	0.623	157	−9.5	NA	104	−8.2	NA	Oparil et al. (28)
	100	None	138	55	0.5	153.3	−11.3	13.39	100.6	−8.7	7.52	Kassler-Taub et al. (27)
		Forced	322	54.1	0.584	152	−9.8	NA	99.9	−8.7	NA	Bakris et al. (21)
			121	57	0.47	162.5	−16	16.4	100.67	−10	9.4	Chung et al. (33)
			200	51.3	0.6	155	−13.4	12.63	103.6	−11.5	8.3	Giles et al. (12)†
			545	55.7	0.575	157.4	−12.8	NA	101.6	−9.7	NA	Hedner et al. (34)
			304	55.1	0.589	152.2	−10.1	NA	100.2	−9.1	NA	Vidt et al. (23)
			103	55	0.62	148	−7.3	18.27	95	−6.7	11.16	White et al. (35)
		Elective	123	55.1	0.63	NA	NA	NA	102.2	−8.6	8.3	Ikeda et al. (30)*
Olmesartan	20	None	145	52.4	0.669	157	−11.3	NA	104	−11.5	NA	Oparil et al. (28)
	40	Forced	199	52.2	0.63	155.4	−13.9	12.6	103.5	−11.7	8.28	Giles et al. (12)
Telmisartan	40	None	71	49.2	0.52	151.1	−17.6	NA	97.2	−11.7	NA	Chen et al. (36)
			57	58	0.67	161.9	−14.2	13.59	100.8	−8.6	7.55	Mallion et al. (31)
			75	51	0.56	153.2	−12.2	14.72	100.7	−10.7	8.66	McGill (37)
			72	54.6	0.69	155.2	−11.6	13.58	100.8	−9.3	7.64	Smith et al. (38)
	80	None	54	57	0.65	164.2	−15.9	13.23	101.8	−9.7	8.08	Mallion et al. (31)
			77	51	0.56	153.2	−15.4	14.92	100.7	−11.5	8.77	McGill (37)
			30	51.1	0.6	167.4	−27.7	NA	102.2	−12.4	NA	Nalbantgil et al. (39)
			72	54.4	0.57	153.7	−11.8	13.58	100	−9.7	7.64	Smith et al. (38)*
	120	None	73	53.2	0.66	151.9	−10	12.82	100.2	−8.8	7.69	Smith et al. (38)*
	160	None	75	53.4	0.68	154.2	−11.9	12.99	100.5	−8.6	7.79	Smith et al. (38)*
Valsartan	40	None	127	55	0.57	153.7	−11.8	11.95	99.2	−10.1	7.55	Philipp et al. study 1 (40)*
	80	None	94	54.1	0.49	NA	NA	NA	100.8	−7	8.5	Monterroso et al. (32)
			142	51.7	0.577	155	−8.4	NA	104	−7.9	NA	Oparil et al. (28)
			124	53.1	0.45	153.2	−12.9	11.8	99.2	−9.7	7.46	Philipp et al. study 1 (40)
			58	56	0.66	152.4	−11.2	12.57	99	−10.5	8.15	Pool et al. (41)
	160	None	666	55.3	0.52	160.2	−15.7	13.32	101.3	−10.8	8.43	Mallion et al. (42)
			1884	55.2	0.553	158.6	−12.97	16	99.81	−10.69	9.59	Parati (43)
			128	53	0.54	152	−15.1	11.99	98.9	−11	7.58	Philipp et al. study 1 (40)
			207	56.8	0.44	155.6	−20.2	13.96	98.9	−13.3	9.06	Philipp et al. study 2 (40)
			166	52.2	0.52	149.6	−14.5	12.63	98.9	−11.7	8.37	Pool et al. (41)
			59	55.1	0.49	154.7	−15.5	12.67	99.1	−11	8.22	Pool et al. (41)
		Forced	551	54.9	0.567	157	−13.8	NA	101.4	−10.5	NA	Hedner et al. (34)
	320	None	128	56.8	0.52	154.6	−15.7	11.99	99.3	−13.4	7.58	Philipp et al. study 1 (40)
			208	56.7	0.52	157.5	−19.8	13.99	99.1	−13.3	9.09	Philipp et al. study 2 (40)
			170	52.5	0.55	149.5	−13.7	12.78	99	−11.3	8.47	Pool et al. (41)
			60	56.8	0.52	153.4	−16.5	12.55	98.9	−11.3	8.13	Pool et al. (41)
		Forced	197	52.2	0.66	154.3	−14.8	12.17	103.3	−12.4	7.88	Giles et al. (12)
			455	52.4	0.62	154.2	−12.8	13.86	100.3	−9.7	8.75	Oparil et al. (44)
			1873	54.6	0.565	159.19	−16.1	15.6	99.88	−11.97	9.51	Parati (43)

* Data from all study arms are used in the model for imputing missing SDs, even if the study arm contains a dose for which the treatment effect is not estimated. Such arms are indicated here.

† Losartan 100 mg is given in 50 mg bid. Data on file was also used from Parati (43). SBP, systolic blood pressure; DBP, diastolic blood pressure; Prop., proportion.

### Quantitative data synthesis

**Figure 3:**
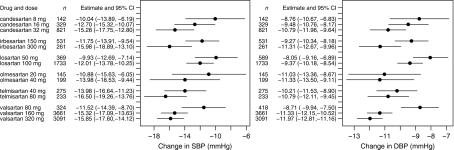
Plot of mean change from baseline SBP and DBP by drug and dose. The number of individuals randomised (*n*) is shown, along with the estimates and 95% CI of the mean change in BP

Figure 3 shows the mean change in SBP and DBP by drug and dose. The results show a dose–response relationship for all ARBs. In particular, a large change in response is noted for valsartan when increasing from 80 to 160 mg and above. Mean change in SBP for valsartan 80, 160 and 320 mg increased from −11.52 mmHg (95% CI: −14.39, −8.70) to −15.32 mmHg (95% CI: −17.09, −13.63) to a further −15.85 mmHg (95% CI: −17.60, −14.12). For DBP, this increase was −8.71 mmHg (95% CI: −9.94, −7.50) to −11.33 mmHg (95% CI: −12.15, −10.52) to a further −11.97 mmHg (95% CI: −12.81, −11.16).

**Figure 4:**
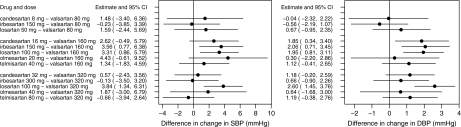
Plot of indirect comparisons of mean change from baseline, and 95% CI, of SBP and DBP by drug and dose. Positive numbers indicate that valsartan is superior to the comparator, negative numbers that valsartan is inferior

Figure 4 shows the indirect comparisons of mean change from baseline in SBP and DBP by drug and dose. Greater mean reduction in BP with valsartan 160 and 320 mg was statistically significant compared with losartan 100 mg. Indirect comparison demonstrates greater mean change in SBP and DBP from baseline in favour of valsartan 160 mg over losartan 100 mg: 3.31 mmHg (95% CI: 0.86, 5.79) and 1.95 mmHg (95% CI: 0.81, 3.11). No significant difference in BP reduction is seen for valsartan 80 mg compared with losartan 50 mg: the difference in the mean change in SBP is 1.59 mmHg (95% CI: −2.44, 5.69) and for DBP is 0.67 mmHg (95% CI: −0.95, 2.35). Irbesartan 150 mg is less effective in reducing SBP and DBP than valsartan 160 mg, with differences in the mean change in BP of 3.56 mmHg (95% CI: 0.77, 6.38) and 2.06 mmHg (95% CI: 0.71, 3.45). Similarly, candesartan 16 mg is less effective in reducing DBP than valsartan 160 mg, with a difference in mean change in DBP of 1.85 mmHg (95% CI: 0.34, 3.40). All other ARBs demonstrate comparable efficacy across dosing ranges.

The estimated value for the meta-regression parameter β is −0.33 mmHg (95% CI: −0.49, −0.17) for SBP and −0.14 mmHg (95% CI: −0.30, 0.01) for DBP. This is interpreted as an average increase of 1 mmHg in the study mean baseline BP leads to an increase of 0.33 mmHg in SBP and 0.14 mmHg in DBP reduction.

## Discussion

Previous similar meta-analyses have failed to compare the antihypertensive efficacy of valsartan 320 mg with other ARBs. In 2000, Conlin et al. (14) performed a meta-analysis comparing BP reduction among ARBs. Main inclusion criteria were prospective; double-blind RCTs with placebo run in of 4–5 weeks; patient population with mild-to-moderate hypertension (DBP: 95–115 mmHg) considered representative of general hypertensive population; clinical measurement of BP using cuff/mercury sphygmomanometer; doses recommended in USA, Japan and Europe and study duration 8–12 weeks. The following ARBs and doses were included for analysis: valsartan 80 and 160 mg, losartan 50 and 100 mg, irbesartan 150 and 300 mg and candesartan 8 and 16 mg. This is the most recent meta-analysis considering valsartan 160 mg, but not 320 mg. From October 1998, 43 trials (*n*= 11,281) demonstrated no clinically meaningful differences in antihypertensive effect. The authors noted that dose titration (forced and elective) resulted in only a modest incremental reduction in DBP compared with starting dose, suggesting a relatively flat dose–response curve.

In 2005, Baguet et al. (15) performed a similar meta-analysis comparing the efficacy across different antihypertensive drug classes (β-blockers, diuretics, calcium channel antagonists, angiotensin-converting enzyme and ARBs). Included studies were in adults over 18 years with slight-to-moderate hypertension (SBP: 140–179 mmHg and/or DBP: 90–109 mmHg). RCTs were quality graded according to Jadad specifications. Drug combinations were not included in analysis. The ARBs considered were candesartan cilexetil 8 and 16 mg, irbesartan 150 and 300 mg, losartan 50 and 100 mg and valsartan 80 mg used in monotherapy, at fixed dose or dose increase. Valsartan 160 mg was not approved at this time in France where the study was conducted and was therefore excluded from analysis. Between publication period 1973 to 2004, 72 trials were analysed (*n*= 9094). Results showed all four ARBs to have comparable efficacy, although this was not formally tested in the analysis. Again, comparison between fixed dose and dose increase regimes did not show greater efficacy for dose increase.

The same authors updated and expanded the analysis in 2007 to include more drugs in each class and added the class renin inhibitors (16). Again, valsartan 160 and 320 mg were not evaluated. Similar inclusion criteria were applied across studies. The following ARBs were compared: candesartan 8 and 16 mg, irbesartan 150 and 300 mg, losartan 50 and 100 mg, olmesartan 20 and 40 mg, telmisartan 40 and 80 mg and valsartan 80 mg, with comparable results.

Methodologically, the Baguet et al. (15) and Conlin et al. (14) meta-analyses use similar approaches, where the overall change in BP is found from the weighted average of the estimates from the studies. However, the weights are the sample sizes and this has an implied assumption of homogeneous variance of the underlying population, this is a fixed-effect meta-analysis. We use the inverse of the variances of the treatment estimates, which is a more common meta-analysis modelling approach and use random-effects meta-analysis. We achieve this by imputing missing variances. Like the meta-analyses of Baguet et al. and Conlin et al., our analysis synthesises within study arm, but not randomised comparisons within study. This was done because not all studies use a common comparator and we did not want to restrict our analysis to only studies where this occurred. Relative changes between study arms are generally more homogeneous across studies than absolute measures from separate study arms. However, in our case, study patient populations and study designs are similar and heterogeneity across trial arms in the change from baseline BP is small.

The studies used in the analysis are all RCTs. However, the study-level characteristics across studies have not been randomised, so meta-analysis is observational in nature. Meta-regression finds a relationship between the mean change in BP and study-level characteristics (in this case the baseline BP). BP reduction is generally larger in patients with a higher baseline BP. The relationship of baseline BP to change in BP at the study level may not be the same as this relationship for individual patients within trials (17). In practice, other studies have shown that baseline BP is a strong predictor of efficacy. This is a generic issue with meta-regression at a study level and individual data are needed to quantify relationships at an individual patient level.

Treatments were compared across doses as defined in Table 1. At present, a high dose of losartan corresponding to valsartan 320 mg is not available. However, in the analysis, the highest two dose of valsartan (160 and 320 mg) are compared with the highest available dose of losartan (100 mg). Giles et al. (12) forced titrated losartan and showed an increase in BP reduction from 100 mg administered once daily at 8 weeks and 50 mg twice daily at 12 weeks. We took the losartan 50 mg twice daily dose to be the 100 mg daily dose in our analysis, which will confer an improvement in the estimation of the effect of losartan 100 mg in the meta-analysis.

The current study is the first to evaluate indirect comparisons of ARBs considering high-dose valsartan and the results demonstrate a dose–response for all ARBs, with a particularly strong response in valsartan.

Generalisation of the results is limited by the inclusion and exclusion criteria applied. The analysis is further limited by scarcity of studies available for valsartan at high doses. Results are confined to monotherapy, whereas many patients in clinical practice receive combination therapy.

## Conclusion

This meta-analysis demonstrates that valsartan at doses of 160 and 320 mg is more effective in reducing BP than losartan at the 100 mg dose. At comparable doses, valsartan achieves comparable antihypertensive efficacy to the other ARBs. Findings confirm that valsartan has a strong dose–response relationship when increasing from 80 to 160 mg and 320 mg and that further head-to-head trial are warranted. The clinical application of these results should take into consideration the limitations discussed in this analysis.

## Author contributions

Richard Nixon developed the statistical methods and performed the analysis; he contributed to developing the literature search strategy, interpreting the results and writing the manuscript. Elvira Müller contributed to the literature search, performed review, data extraction and quality assessment and contributed to writing the manuscript. Adam Lowy contributed to developing the literature search strategy, providing clinical input and contributed ideas for the manuscript. Heather Falvey provided interpretation of results, discussion on this interpretation and was involved in writing the manuscript.

## Appendix

### Missing data model (SD of the change from baseline BP)

Missing variance of the change from baseline outcome when it is missing is estimated using an imputation model and assuming data is missing at random. The log of the summary statistics (i) SD of BP at baseline S_1_, (ii) SD of BP at follow-up S_2_ and (iii) SD of the change in BP between baseline and follow-up S_d_ are assumed to be sampled from a tri-variate normal distribution.

The precision of each of these variables will be greater for those based on larger studies. The expected value and variance of the maximum likelihood estimate of a variance are  and  respectively, where  is the true variance. Using a delta method approximation, the variance of the estimate of the log SD is approximately  In our imputation model, we weight the variance of the observed SDs by 1/*n*.

Furthermore, we exploit knowledge about the relationship between the SDs. If  is the correlation between baseline and follow-up BP measures, which we assume is the same in each arm, *i*, then

(1)

The imputation model for the baseline and follow-up SDs is

(2)

and for the change from baseline is

(3)

### Meta-analysis model

*i* indexes the treatment arm across all studies, *k*(*i*) indexes the treatment and dose used. For example, *k*(*i*) takes the value one for candesartan 8 mg. *d*_*i*_ is the change in BP from baseline to follow-up; *s*_di_ is the SD of the change in BP between baseline and follow-up; *n*_i_ is the number of patients at baseline; *m*_1*i*_ is the mean baseline BP, re-centred about the mean. We use a random-effects meta-regression model for the change in BP. Although the patients in each study are randomised, differences between the characteristics of the patients used in the different studies are not randomised and these may influence the treatment effect. Meta-regression can adjust for this. We can explore how difference in baseline blood pressure influences the treatment.

(4)

where  is the treatment random effect (mean difference in BP from baseline to follow-up) for treatment arm *i*. β is the effect of mean baseline BP on the change in BP. We assume this is the same effect across all treatment types.  is the overall treatment effect of each type of treatment.  is the between study heterogeneity. We assume this heterogeneity is the same for all types of treatment.

Model fitting is performed in WinBUGS to facilitate imputing the missing values and carrying this uncertainty through into the meta-analysis models in one step. Each model used three chains, was burnt in for 5000 iterations per chain, then run further to sample 20,000 values. All models mixed well around their stationary distributions.

## Supporting information

**Appendix S1** Study Quality Evaluation Sheet.

## References

[1] Kearney PM, Whelton M, Reynolds K, Muntner P, Whelton PK, He J (2005). Global burden of hypertension: analysis of worldwide data. Lancet.

[2] Whitworth JA (2003). World Health Organization (WHO)/International Society of Hypertension (ISH) statement on management of hypertension. J Hypertens.

[3] Hyman DJ, Pavlik VN (2001). Characteristics of patients with uncontrolled hypertension in the United States. N Engl J Med.

[4] Cohn JN, Tognoni G (2001). A randomized trial of the angiotensin-receptor blocker valsartan in chronic heart failure. N Engl J Med.

[5] Pfeffer MA, McMurray JJ, Velazquez EJ (2003). Valsartan, captopril, or both in myocardial infarction complicated by heart failure, left ventricular dysfunction, or both. N Engl J Med.

[6] Julius S, Kjeldsen SE, Weber M (2004). Outcomes in hypertensive patients at high cardiovascular risk treated with regimens based on valsartan or amlodipine: the VALUE randomised trial. Lancet.

[7] Halpern M, Falvey H (2007). The Impact of Valsartan vs. Losartan Therapy on 24-Hour Ambulatory BP and Long-Term Cardiovascular Outcomes. Poster No. 1308, ESH – 17th European Meeting on Hypertension.

[8] Wogen J, Kreilick CA, Livornese RC, Yokoyama K, Frech F (2003). Patient adherence with amlodipine, lisinopril, or valsartan therapy in a usual-care setting. J Manag Care Pharm.

[9] Verdecchia P, Angeli F (2004). Assessment of the optimal daily dose of valsartan in patients with hypertension, heart failure, or both. Clin Ther.

[10] Jan SA, Patel JV, Welz J, Ishak P (2005). A retrospective database analysis of prescribing patterns for specific angiotensin receptor blockers. Drug Benefits Trends.

[11] Pool JL, Guthrie RM, Littlejohn TW (1998). Dose-related antihypertensive effects of irbesartan in patients with mild-to-moderate hypertension. Am J Hypertens.

[12] Giles TD, Oparil S, Silfani TN, Wang A, Walker JF (2007). Comparison of increasing doses of olmesartan medoxomil, losartan potassium, and valsartan in patients with essential hypertension. J Clin Hypertens (Greenwich).

[13] Moher D, Cook DJ, Jadad AR (1999). Assessing the quality of reports of randomised trials: implications for the conduct of meta-analyses. Health Technol Assess.

[14] Conlin PR, Spence JD, Williams B (2000). Angiotensin II antagonists for hypertension: are there differences in efficacy?. Am J Hypertens.

[15] Baguet JP, Robitail S, Boyer L, Debensason D, Auquier P (2005). A meta-analytical approach to the efficacy of antihypertensive drugs in reducing blood pressure. Am J Cardiovasc Drugs.

[16] Baguet JP, Legallicier B, Auquier P, Robitail S (2007). Updated meta-analytical approach to the efficacy of antihypertensive drugs in reducing blood pressure. Clin Drug Investig.

[17] Thompson SG, Higgins JP (2002). How should meta-regression analyses be undertaken and interpreted?. Stat Med.

[18] Reif M, White WB, Fagan TC (1998). Effects of candesartan cilexetil in patients with systemic hypertension. Candesartan Cilexetil Study Investigators. Am J Cardiol.

[19] Andersson OK, Neldam S (1998). The antihypertensive effect and tolerability of candesartan cilexetil, a new generation angiotensin II antagonist, in comparison with losartan. Blood Press.

[20] Zuschke CA, Keys I, Munger MA (1999). Candesartan cilexetil: comparison of once-daily versus twice-daily administration for systemic hypertension. Candesartan Cilexetil Study Investigators. Clin Ther.

[21] Bakris G, Gradman A, Reif M (2001). Antihypertensive efficacy of candesartan in comparison to losartan: the CLAIM study. J Clin Hypertens (Greenwich, Conn).

[22] Kloner RA, Weinberger M, Pool JL (2001). Comparative effects of candesartan cilexetil and amlodipine in patients with mild systemic hypertension. Comparison of Candesartan and Amlodipine for Safety, Tolerability and Efficacy (CASTLE) Study Investigators. Am J Cardiol.

[23] Vidt DG, White WB, Ridley E (2001). A forced titration study of antihypertensive efficacy of candesartan cilexetil in comparison to losartan: CLAIM Study II. J Hum Hypertens.

[24] Kochar M, Guthrie R, Triscari J, Kassler-Taub K, Reeves RA (1999). Matrix study of irbesartan with hydrochlorothiazide in mild-to-moderate hypertension. Am J Hypertens.

[25] Fogari R, Ambrosoli S, Corradi L (1997). 24-hour blood pressure control by once-daily administration of irbesartan assessed by ambulatory blood pressure monitoring. Irbesartan Multicenter Investigators’ Group. J Hypertens.

[26] Gradman AH, Schmieder RE, Lins RL, Nussberger J, Chiang Y, Bedigian MP (2005). Aliskiren, a novel orally effective renin inhibitor, provides dose-dependent antihypertensive efficacy and placebo-like tolerability in hypertensive patients. Circulation.

[27] Kassler-Taub K, Littlejohn T, Elliott W, Ruddy T, Adler E (1998). Comparative efficacy of two angiotensin II receptor antagonists, irbesartan and losartan in mild-to-moderate hypertension. Irbesartan/Losartan Study Investigators. Am J Hypertens.

[28] Oparil S, Williams D, Chrysant SG, Marbury TC, Neutel J (2001). Comparative efficacy of olmesartan, losartan, valsartan, and irbesartan in the control of essential hypertension. J Clin Hypertens (Greenwich, Conn).

[29] Ali G, Kamili MMA, Kumar M (2001). Efficacy & tolerability of losartan compared with amlodipine in the treatment of essential hypertension. JK Pract.

[30] Ikeda LS, Harm SC, Arcuri KE, Goldberg AI, Sweet CS (1997). Comparative antihypertensive effects of losartan 50 mg and losartan 50 mg titrated to 100 mg in patients with essential hypertension. Blood Press.

[31] Mallion JM, Siche JP, Lacourciere Y (1999). ABPM comparison of the antihypertensive profiles of the selective angiotensin II receptor antagonists telmisartan and losartan in patients with mild-to-moderate hypertension. The Telmisartan Blood Pressure Monitoring Group [see comments]. J Hum Hypertens.

[32] Monterroso VH, Chavez VR, Carbajal ET (2000). Use of ambulatory blood pressure monitoring to compare antihypertensive efficacy and safety of two angiotensin II receptor antagonists, losartan and valsartan. Adv Ther.

[33] Chung O, Hinder M, Sharma AM (2000). Comparison of the efficacy and safety of losartan (50–100 mg) with the T-type calcium channel blocker mibefradil (50–100 mg) in mild to moderate hypertension. Fundam Clin Pharmacol.

[34] Hedner T, Oparil S, Rasmussen K (1999). A comparison of the angiotensin II antagonists valsartan and losartan in the treatment of essential hypertension. Am J Hypertens.

[35] White WB, Sica DA, Calhoun D, Mansoor GA, Anders RJ (2002). Preventing increases in early-morning blood pressure, heart rate, and the rate-pressure product with controlled onset extended release verapamil at bedtime versus enalapril, losartan, and placebo on arising. Am Heart J.

[36] Chen JH, Cheng JJ, Chen CY (2004). Comparison of the efficacy and tolerability of telmisartan 40 mg vs. enalapril 10 mg in the treatment of mild-to-moderate hypertension: a multicentre, double-blind study in Taiwanese patients. Int J Clin Pract Suppl.

[37] McGill JB (2001). Angiotensin II receptor antagonist plus a thiazide diuretic is more efficacious for treating hypertension than either drug alone. Blood Press Monit.

[38] Smith DHG, Neutel JM, Morgenstern P (1998). Once-daily telmisartan compared with enalapril in the treatment of hypertension. Adv Ther.

[39] Nalbantgil I, Nalbantgil S, Ozerkan F (2004). The efficacy of telmisartan compared with perindopril in patients with mild-to-moderate hypertension. Int J Clin Pract Suppl.

[40] Philipp T, Smith TR, Glazer R (2007). Two multicenter, 8-week, randomized, double-blind, placebo-controlled, parallel-group studies evaluating the efficacy and tolerability of amlodipine and valsartan in combination and as monotherapy in adult patients with mild to moderate essential hypertension. Clin Ther.

[41] Pool JL, Glazer R, Weinberger M, Alvarado R, Huang J, Graff A (2007). Comparison of valsartan/hydrochlorothiazide combination therapy at doses up to 320/25 mg versus monotherapy: a double-blind, placebo-controlled study followed by long-term combination therapy in hypertensive adults. Clin Ther.

[42] Mallion JM, Carretta R, Trenkwalder P (2003). Valsartan/hydrochlorothiazide is effective in hypertensive patients inadequately controlled by valsartan monotherapy. Blood Press Suppl.

[43] Parati G, Mengden T, Brudi P, Kandra A, Di Giovanni R, Asmar R (2006). Efficacy and safety of valsartan 160 mg and 320 mg od as monotherapy in mild to moderate essential hypertension. J Hypertens.

[44] Oparil S, Yarows SA, Patel S, Fang H, Zhang J, Satlin A (2007). Efficacy and safety of combined use of aliskiren and valsartan in patients with hypertension: a randomised, double-blind trial. Lancet.

