# Supplementary material for: Valsartan vs. other angiotensin II receptor blockers in the treatment of hypertension: a meta-analytical approach
Source: Int J Clin Pract. 2009 May;63(5):766–75. doi: 10.1111/j.1742-1241.2009.02028.x (PMC2779985; doi:10.1111/j.1742-1241.2009.02028.x)
Supplement: Supplementary file 1 [file ijcp0063-0766-SD1.pdf]

## Online Supplementary Material: Study Quality Evaluation Sheet.

Highlighted fields apply to this article.
First Author:

Year:

Criteria

Selection Bias (*before* allocation)

*Randomisation*
True: Computer-generated, Random Number Generation, Coin Toss (2) Die Toss (3)
Quasi: Alternation, Case record numbers, Birth dates, week days

---

*Allocation Concealment*
Adequate: Computer allocation (e.g. central office), On-site computer, Pre-coded containers (identical), Serially numbered envelopes
Inadequate: Open Methods, non-opaque envelopes, odd/even number medical records, DOB, days week, Alternation, Unspecified tables, lists, random numbers, sealed envelopes

---

Performance Bias (*during* treatment)
Blinding*
*If a study is 'double-blind' it will be assumed that the participant and HC provider are blinded.
If the study is said to be 'blinded' we will exclude it.

Participants

HC Provider

Only additional criteria:
Outcome Assessor

Data Analyst

---

Attrition Bias (*after* allocation and inclusion)

Reports withdrawals from trial

Per Protocol Analysis (second choice)

Intention To Treat Analysis (preferred)

---

Detection Bias

Blinding of outcome assessors?

Validity of Outcome Tools (i.e. information on outcome tools)

*Selective Outcome Reporting*

---

*Study Included*

*Study Excluded*

*Name of Assessor*

*Comment*
